# Supplementary material for: A single-cell analysis reveals tumor heterogeneity and immune environment of acral melanoma
Source: Nat Commun. 2022 Nov 25;13:7250. doi: 10.1038/s41467-022-34877-3 (PMC9700682; doi:10.1038/s41467-022-34877-3)
Supplement: Supplementary file 1 — Supplementary Information [file 41467_2022_34877_MOESM1_ESM.pdf]

Supplementary information

A single-cell analysis reveals tumor heterogeneity and immune environment of acral melanoma

Supplementary Figures

Supplementary figure 1

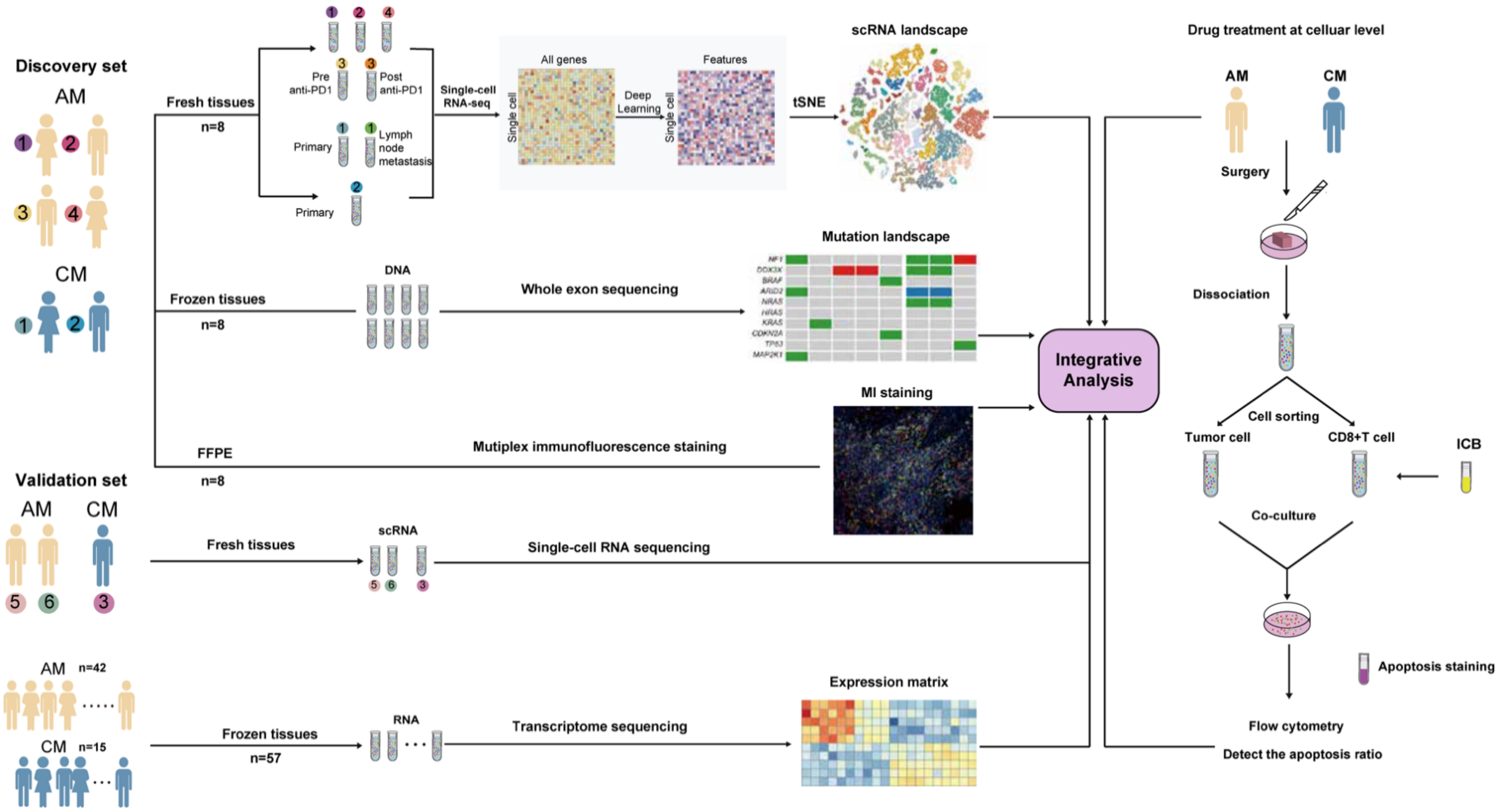

Supplementary figure 1. Schematic representation of the experimental strategy. Summary of study design, including the designs of scRNA sequencing, whole-exon sequencing, multiplex immunofluorescence staining, transcriptome sequencing and drug treatment experiment at the cellular level.

Supplementary figure 2

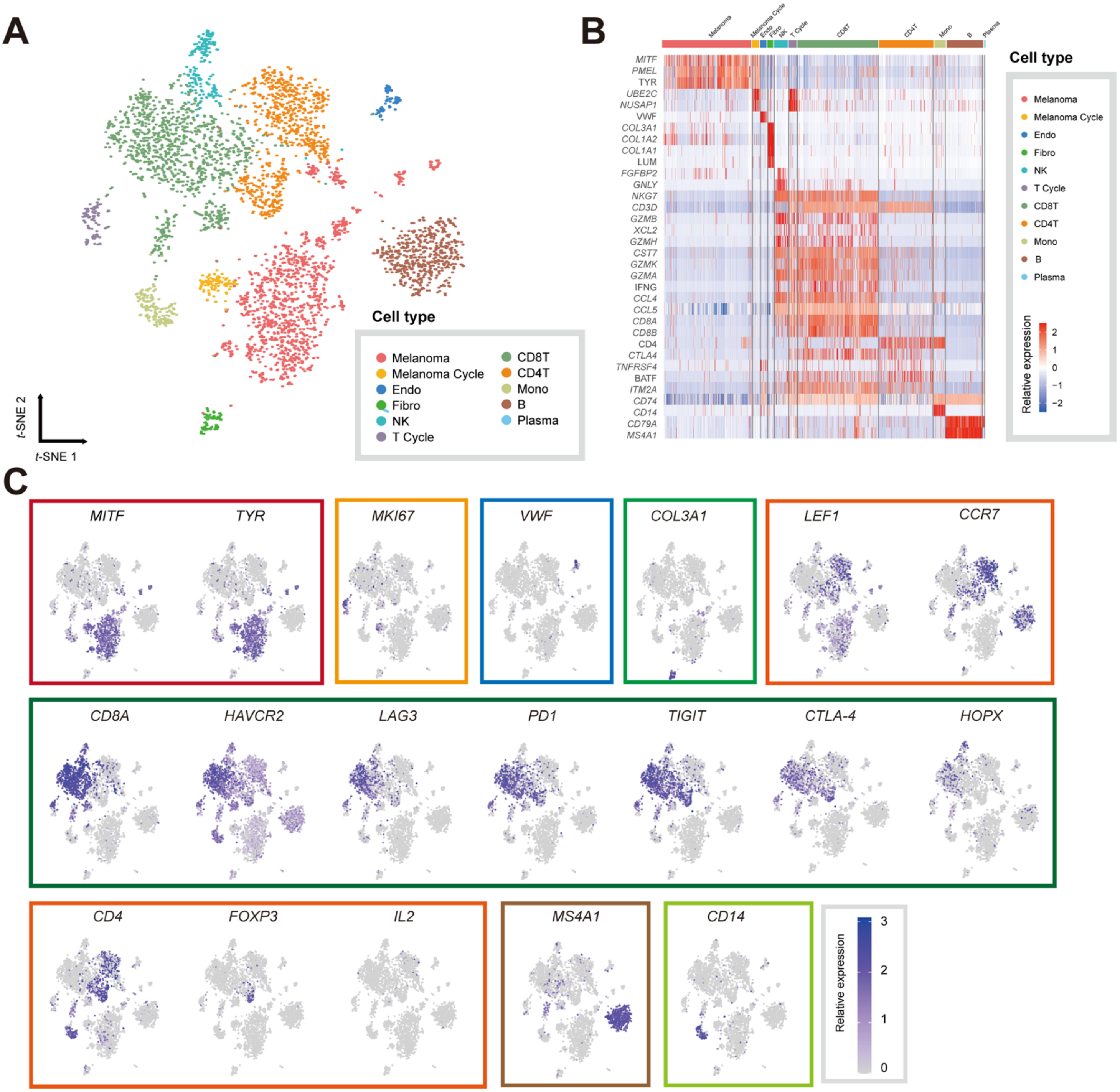

Supplementary figure 2. The identified marker genes obtained from our single-cell atlas mapping in the GSE72056 dataset.

(A) T-distributed stochastic neighbor embedding (t-SNE) plot, showing the annotation and color codes for cell types in the external dataset of melanoma ecosystem.

(B) Heatmap showing the expression of marker genes in the cells. The top bars label the clusters corresponding to specific cell types. (Fib, Fibroblast cell. Endo, Endothelial cell, Mono Monocyte.)

(C) T-distributed stochastic neighbor embedding (t-SNE) plot, showing the genes relative expression of each cell.

Supplementary figure 3

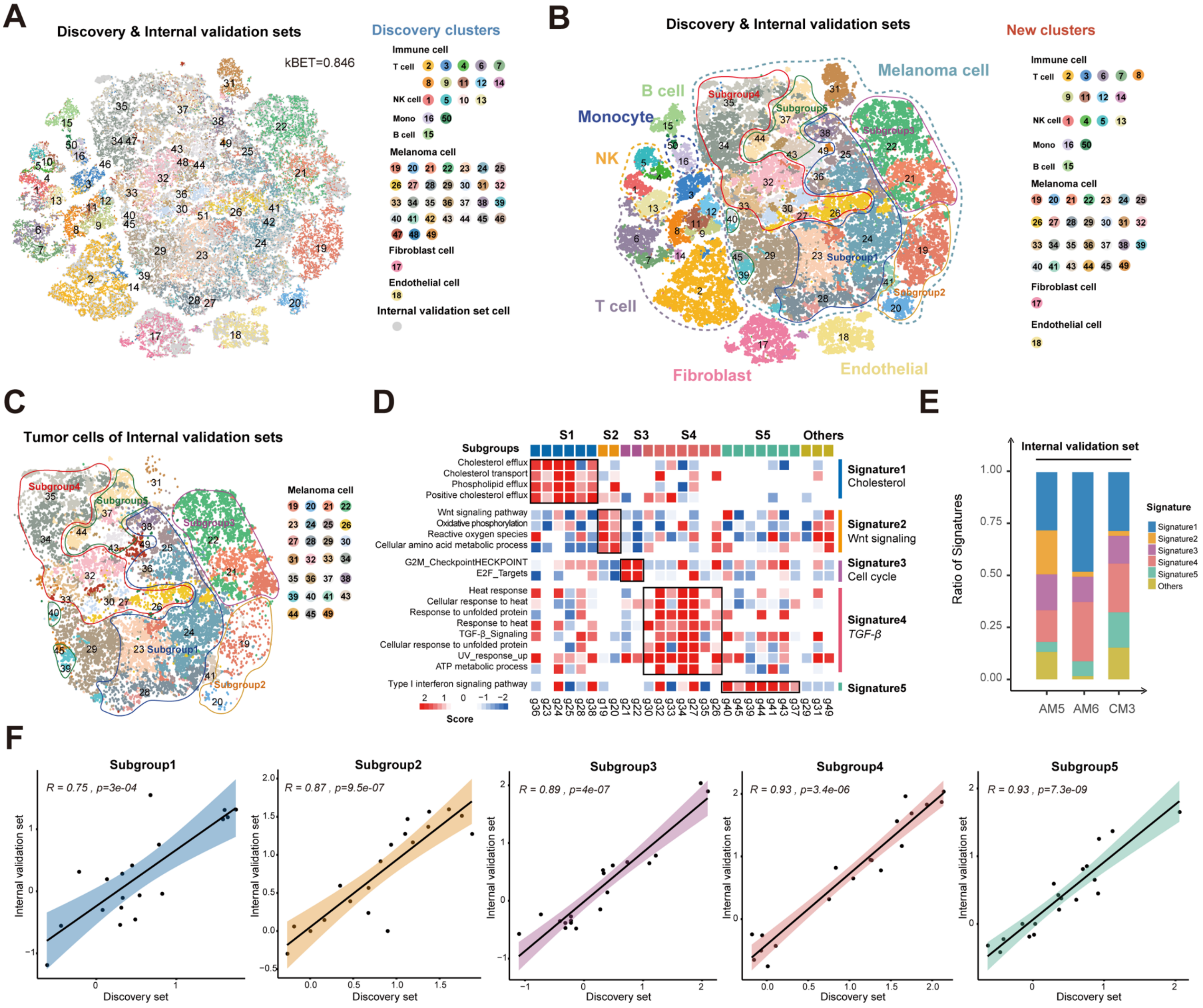

Supplementary figure 3. **scRNA-seq profiling of the acral and cutaneous melanoma environments in the internal validation set.**

(A) t-SNE plot showing the single cell clustering in the integration of discovery, internal validation set, kBET acceptance rate and cell origins by color. (Mono, Monocyte)

(B) t-SNE showing the annotation and color codes for cell types and melanoma cell subgroups in the melanoma ecosystem and cell origins by color of Discovery & Internal validation sets.

(C) t-SNE showing the annotation and color codes for cell types and melanoma cell subgroups in the melanoma ecosystem and cell origins by color of tumor cells in Internal validation sets.

(D) Heatmap showing the expression score by ssGESA in the tumor clusters (5 Signatures) internal validation set, including biological functions and names of related signal pathways. Source data are provided as a Source Data file.

(E) Histogram indicating the proportion of signatures in melanoma of internal validation set samples.

(F) The scatter diagram shows the correlation of pathway scores of two dataset tumor cells in each subgroups, using a two-sided Spearman's correlation test. Error bands represent 95% confidence interval, respectively. Source data are provided as a Source Data file.

Supplementary figure 4

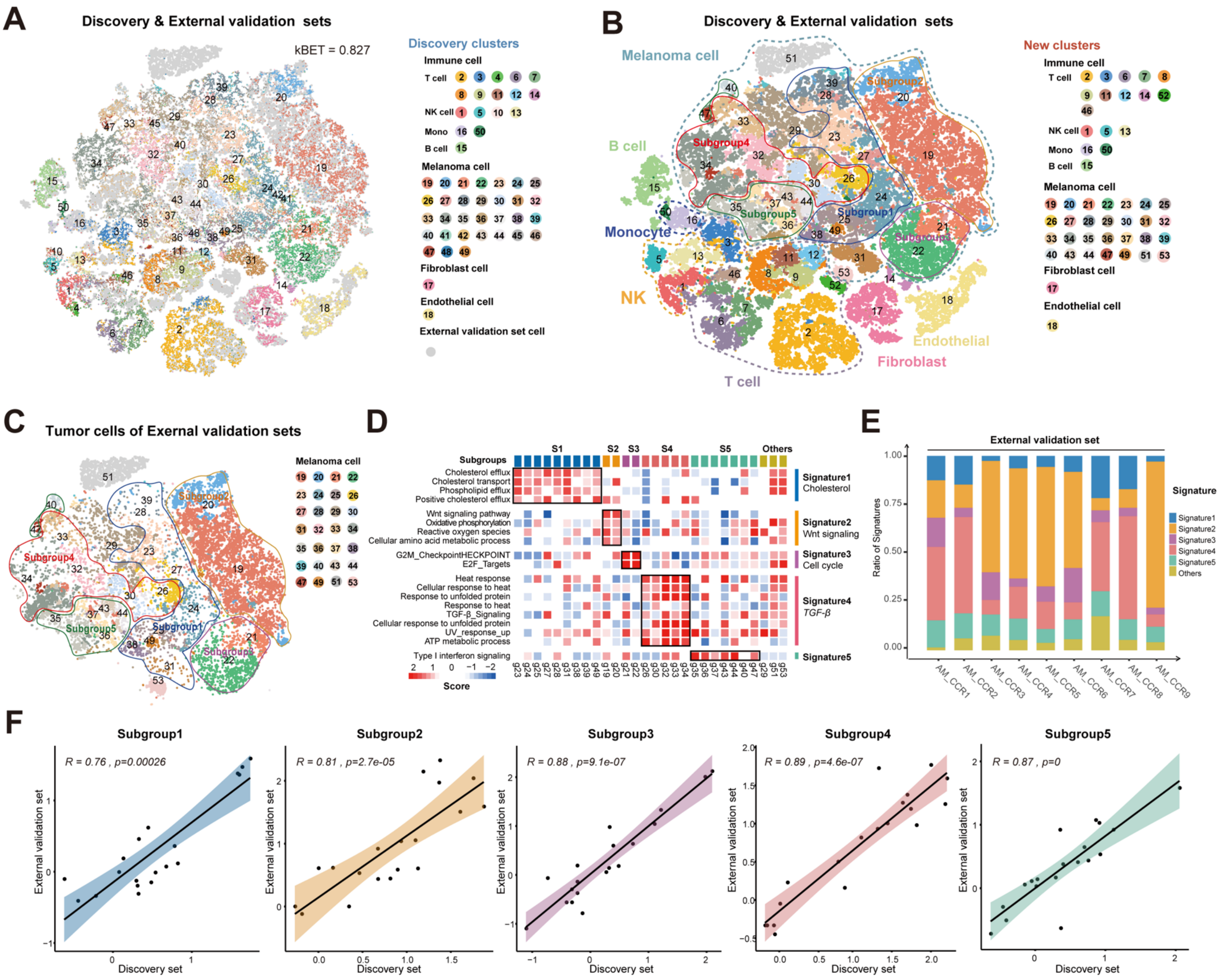

Supplementary figure 4. **scRNA-seq profiling of the acral and cutaneous melanoma environments in the discovery and external validation dataset.**

(A) t-SNE plot showing the single cell clustering in the integration of discovery, external. validation set, kBET acceptance rate and cell origins by color.

(B) t-SNE showing the annotation and color codes for cell types and melanoma cell subgroups in. the melanoma ecosystem and cell origins by color of Discovery & External validation sets. (Mono, Monocyte)

(C) t-SNE showing the annotation and color codes for cell types and melanoma cell subgroups in. the melanoma ecosystem and cell origins by color of tumor cells in external validation sets.

(D) Heatmap showing the expression score by ssGESA in the tumor clusters (5 Signatures) of. external validation set, including biological functions and names of related signal pathways. Source data are provided as a Source Data file.

(E) Histogram indicating the proportion of signatures in melanoma of external validation set. samples.

(F) The scatter diagram shows the correlation of pathway scores of two dataset tumor cells in each subgroups, using a two-sided Spearman's correlation test. Error bands represent 95% confidence interval, respectively. Source data are provided as a Source Data file.

Supplementary figure 5

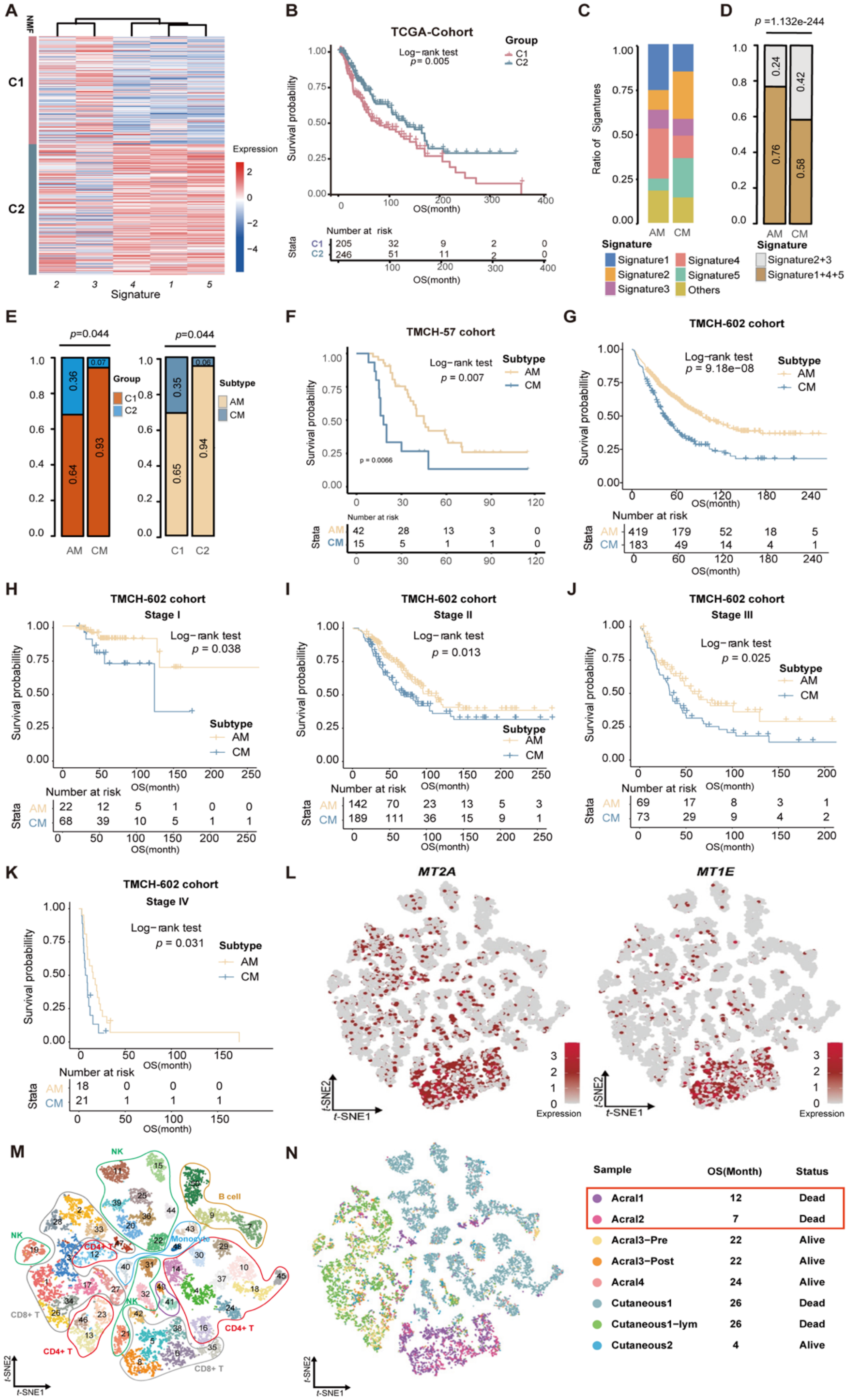

Supplementary figure 5. Association between Signatures and overall survival

(A) Heatmap showing the expression score of 451 TCGA-SKCM patient's bulk-RNA data by ssGESA in the 5 signatures, including 2 clusters of NMF. Source data are provided as a Source Data file.

(B) Kaplan-Meier analysis showing the overall survival rate of 451 TCGA-Cohort patients, characterized by C1 (pink) and C2 (dark green). The numbers of patients and the risk classification are indicated in the figure. Significance was calculated using the log-rank test. Source data are provided as a Source Data file.

(C) Histogram indicating the proportion of signatures in 4 acral samples (Acral1, Acral2, Acral3-pre, Acral4) and 3 cutaneous samples (Cutaneous1, Cutaneous2, Cutaneous1-lym).

(D) Histogram indicating the proportion of signature(2+3) and signature(1+4+5) in 4 acral samples (Acral1, Acral2, Acral3-pre, Acral4) and 3 cutaneous samples (Cutaneous1, Cutaneous2, Cutaneous1-lym) from Discovery scRNA-seq set. Significance was calculated using the two-side, unpaired Fisher's exact test, P-value = 1.132e-244.

(E) Histogram indicating the proportion of NMF clusters and melanoma subtypes in 42 AM and 15 CM samples from TMCH-57 Cohort. Significance was calculated using the two-side, unpaired Fisher's exact test, P-value = 0.044, 0.044, respectively.

(F) Kaplan-Meier analysis showing the overall survival rate of 57 TMCH-Cohort patients, characterized by AM (yellow) and CM (blue). The numbers of patients and the risk classification are indicated in the figure. Significance was calculated using the log-rank test. Source data are provided as a Source Data file.

(G-K) Kaplan-Meier analysis showing the overall survival rate of 602 TMCH-602 Cohort patients, characterized by AM (yellow) and CM (blue), including all patients (G), stage I patients (H), stage II patients (I), stage III patients (J), stage IV patients (K). The numbers of patients and the risk classification are indicated in the figure. Significance was calculated using the log-rank test. Source data are provided as a Source Data file.

(L) T-distributed stochastic neighbor embedding (t-SNE) plot, showing the *MT2A* and *MT1E*. relative expression of each cell.

(M) T-SNE plot showing the clusters of immune cells and cell origins by color, according to. immune cell types

(N) T-SNE plot showing the clusters of immune cells with overall survival information and cell. origins by color, according to samples.

Supplementary figure 6

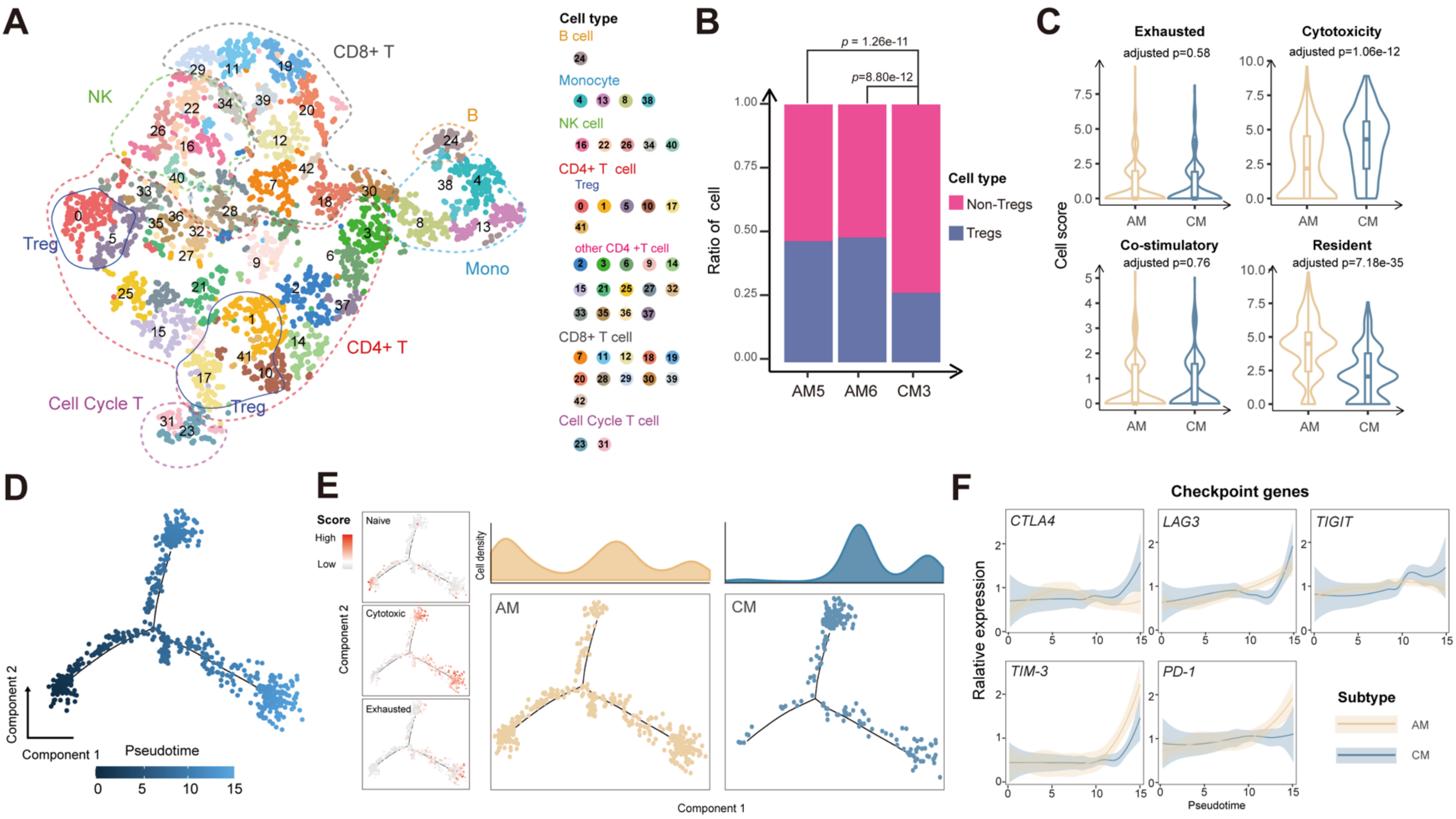

Supplementary figure 6. Analysis of CD8+ T cell transition states for acral melanoma and cutaneous melanoma samples in internal validation dataset.

(A) t-SNE plot showing the clusters of immune cells and cell origins by color, according to immune cell types in the internal validation dataset.

(B) Histogram indicating the proportion of Treg cells for each sample. Significance was calculated using the two-side, unpaired Fisher's exact test.

(C) Violin plot showing the expression of signature genes in CD8 + T cells in AM (yellow; Acral5 and Acral6) and CM (blue; Cutaneous3) samples. Significance was determined by Wilcoxon rank sum test. Box center lines, bounds of the box, and whiskers indicate medians, first and third quartiles, and minimum and maximum values within 1.5×IQR (interquartile range) of the box limits, respectively. Significance was determined using a two-sided, unpaired Wilcoxon rank-sum test relative to AM (n = 549 cells) for CM (n = 216 cells, Exhausted P-value = 0.58, Cytotoxicity P-value = 1.06e-12, Co-stimulatory P-value = 0.76, Resident P-value = 7.18e-35). Source data are provided as a Source Data file.

(D) Pseudotime-ordered analysis of CD8 + T cells from AM and CM samples in the internal validation dataset. T cell subtypes are labeled by colors.

(E) 2D pseudotime plot showing the dynamics of naïve (upper panel), cytotoxic (middle panel) or exhausted signals (lower panel) in CD8 + T cells, AM (left panel) and CM (right panel) in the internal validation dataset. The cell density distribution, by state, is shown at the top of the figure. Source data are provided as a Source Data file.

(F)Two-dimensional plots showing the dynamic expression cytokines genes and checkpoint genes in the internal validation dataset Error bands show local polynomial regression and the 95% confidence interval, respectively.

Supplementary figure 7

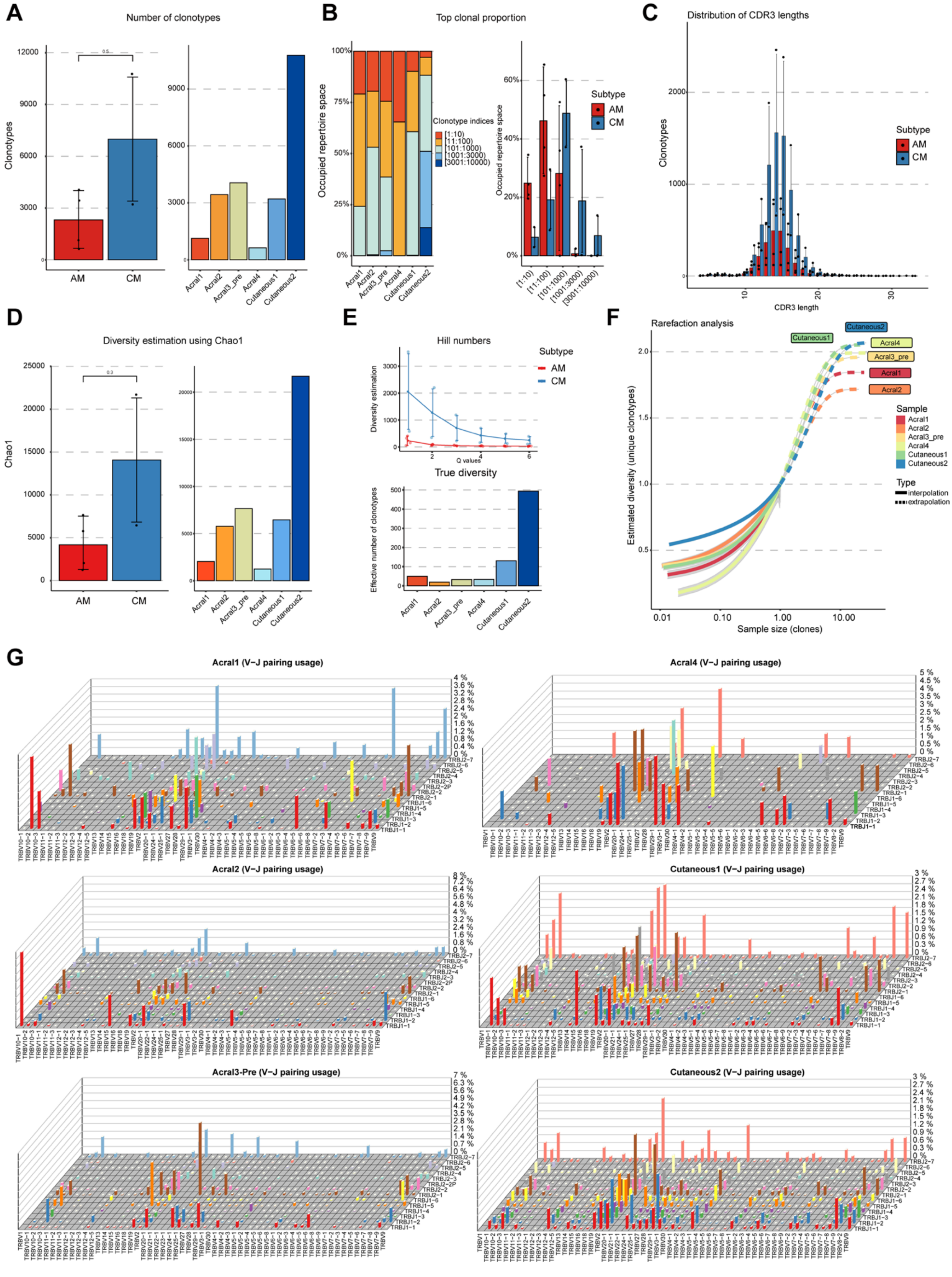

Supplementary figure 7. The characteristics of T cell receptor clonotype and diversity across the acral melanoma and cutaneous melanoma.

(A) The number of T cell clonotypes across melanoma patients, and those of grouping by subtypes AM (n = 4 samples) and CM (n = 2 samples). Data are presented as mean values +/- SEM (left panel).

(B) Distribution of the most abundant clonotypes by patient, and those of AM (n = 4 samples) and CM (n = 2 samples). Data are presented as mean values +/- SEM (right panel).

(C) Distribution of CDR3 length in AM (n = 4 samples) and CM (n = 2 samples). Data are presented as mean values +/- SEM.

(D and E) The diversity of T cell clonotypes across melanoma patients, and those of grouping by subtypes AM (n = 4 samples) and CM (n = 2 samples), evaluating via Chao1, Hill and True diversity. Data are presented as mean values +/- SEM (left panel of D and up panel of E).

(F) The rarefaction analysis of T cell receptor diversity across melanoma patients.

(G) VDJ gene usage of T cell receptor in AM and CM patients.

Supplementary figure 8.

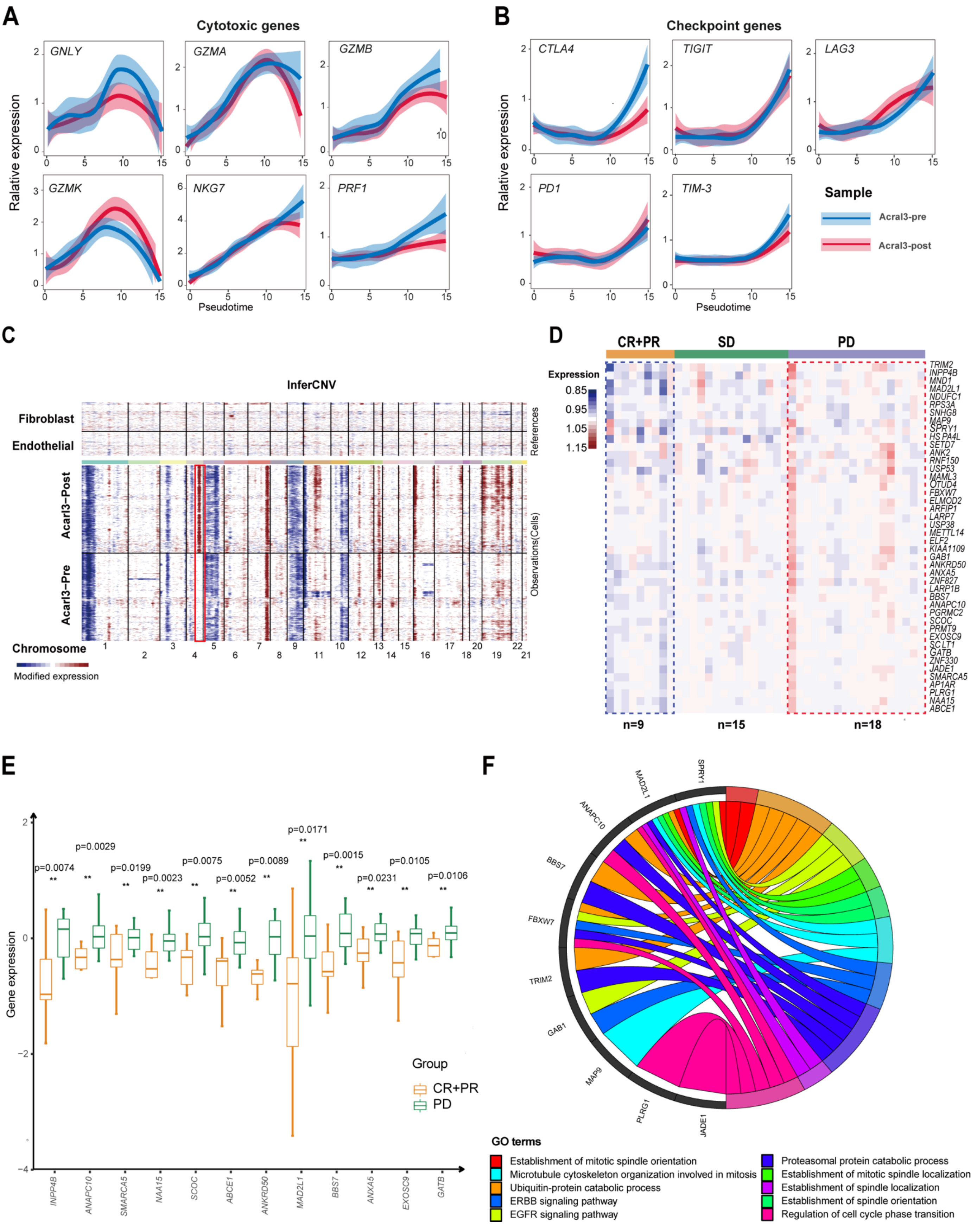

Supplementary figure 8. Differences of copy number variation before and after treatment in an immune-resistant patient with acral melanoma

(A and B) Two-dimensional plots showing the dynamic expression cytokines genes(A) and checkpoint genes (B) during the T cell transitions along the pseudo-time. Error bands show local polynomial regression and the 95% confidence interval, respectively.

(C) Representative CNV heatmaps with hierarchical clustering from inferCNV analysis from a pair of samples (Acral-3 pre and Acral3-post) before and after receiving immunotherapy.

(D) Heatmap showing the changes of 44 gene expression in matching samples received anti-PD1 therapy. PD, progressive disease. SD, stable disease. PR, partial response. CR, complete response.)

(E) Boxplot shows the genes with significant differences in the CR+PR and PD groups. Box center lines, bounds of the box, and whiskers indicate medians, first and third quartiles, and minimum and maximum values within 1.5×IQR (interquartile range) of the box limits, respectively. Significance was determined using a two-sided, unpaired Wilcoxon rank-sum test relative to CR+PR group (n = 9 samples) for PD group (n = 18 samples, P-value are 0.0074, 0.0029, 0.0199, 0.0023, 0.0075, 0.0052, 0.0089, 0.0170, 0.0015, 0.0231, 0.0105, 0.0106, respectively). PD, progressive disease. SD, stable disease. PR, partial response. CR, complete response.)

(F) Top 10 GO terms of 44 genes which were significantly amplified after anti-PD1 treatment.

Supplementary figure 9

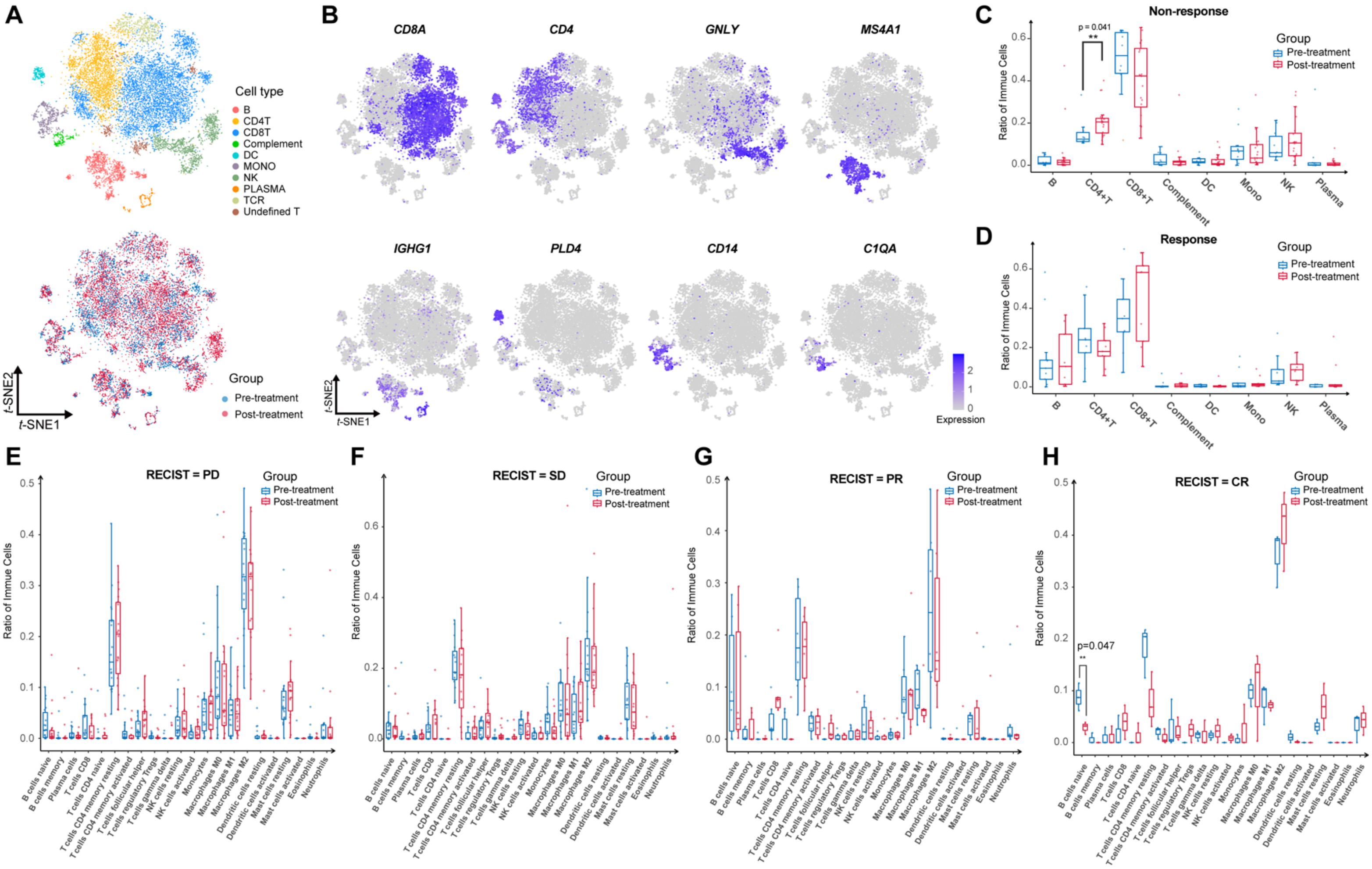

Supplementary figure 9. Differences of immune cells before and after treatment in patient with melanoma

(A) T-distributed stochastic neighbor embedding (t-SNE) plot, showing the annotation and color codes for cell types in the immune cells of melanoma ecosystem and cell origins by color(up panel), treatment origin (down panel). (DC, dendritic cells. Mono, monocytes)

(B) T-distributed stochastic neighbor embedding (t-SNE) plot, showing the genes relative expression of each cell.

(C and D) Boxplots illustrating the fraction of immune cell subtypes in pre- and post- immune therapy treatment cutaneous melanoma patients with non-response (C) and response (D), respectively. Box center lines, bounds of the box, and whiskers indicate medians, first and third quartiles, and minimum and maximum values within 1.5×IQR (interquartile range) of the box limits, respectively. Significance was determined using a two-sided, unpaired Wilcoxon rank-sum test relative to Response pre-trentment (n = 9 samples) for Response post-trentment (n = 9 samples, P-value of CD4+ T cell was 0.041), and Non-response pre-trentment (n = 10 samples) for Non-response post-trentment (n = 20 samples). (DC, dendritic cells. Mono, monocytes) Source data are provided as a Source Data file.

(E-H) Boxplots illustrating the fraction of immune cell subtypes in pre- and post- immune therapy treatment cutaneous melanoma patients with PD (E), SD (F), PR (G) and CR(H), respectively. Significance was calculated using the Wilcoxon rank sum test. Box center lines, bounds of the box, and whiskers indicate medians, first and third quartiles, and minimum and maximum values within 1.5×IQR (interquartile range) of the box limits, respectively. Significance was determined using a two-sided, unpaired Wilcoxon rank-sum test relative to PD group pre-trentment (n =18 samples) for Response post-trentment (n =18 samples), and SD pre-trentment (n = 15 samples) for Non-response post-trentment (n = 15 samples), and PR pre-trentment (n = 6 samples) for Non-response post-trentment (n = 6 samples), and CR pre-trentment (n = 3 samples) for Non-response post-trentment (n = 3 samples, B cell P-value = 0.047). (PD, progressive disease. SD, stable disease. PR, partial response. CR, complete response.) Source data are provided as a Source Data file.

Supplementary figure 10

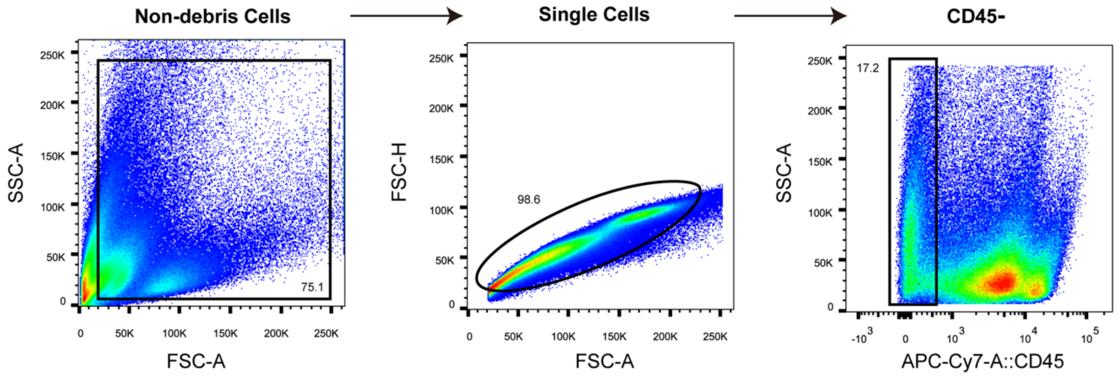

Supplementary figure 10. Flow cytometry gating strategy for detection of melanoma cell apoptosis. Non-debris Cells were identified using FSC (forward scatter) and SSC (side scatter) characteristics as indicated (left panel). Single cells were then gated based on FSC-A and FSC-H (middle panel). The final sort for the melanoma fractions was of single cells from the $CD45^{-}$ compartment (right panel). Percentages reflect the fraction of the (previous) parent population.

Supplementary Table

Supplementary Table 1. Detail clinical and pathological information of Discovery set and Internal validation set

	Samples	Sex	age	subtype	OS (Month)	State	Tumor Size(cm)	Stage	Immune therapy	Site
Discovery set	Acral1	F	56	AM	12	Dead	2*2.5	IIIC	No	Finger
	Acral2	M	61	AM	7	Dead	1.5*1.9	IIIC	No	Sole
	Acral3-pre	M	68	AM	22	Alive	3.5*2.9	IIIC	Yes	Sole
	Acral3-post	M	68	AM	22	Alive	3.5*2.9	IIIC	Yes	Sole
	Acral4	M	58	AM	24	Alive	1.7*1.5	IIIB	No	Sole
	Cutaneous1	M	62	CM	26	Dead	1.3*1.5	IIIC	No	Head
	Cutaneous2	F	80	CM	4	Alive	1*1	IV	No	Face
	Cutaneous1-Lym	M	62	CM	26	Dead	2*1.3	IIIC	No	Neck
Internal validation set	Acral5	M	59	AM	6	Alive	2*3	IIIC	No	Sole
	Acral6	M	70	AM	6	Alive	3*4	IV	No	Sole
	Cutaneous3	M	54	CM	16	Alive	1.5*1.5	IIIC	No	Face
