# Supplementary material for: A single-cell analysis reveals tumor heterogeneity and immune environment of acral melanoma
Source: Nat Commun. 2022 Nov 25;13:7250. doi: 10.1038/s41467-022-34877-3 (PMC9700682; doi:10.1038/s41467-022-34877-3)
Supplement: Supplementary file 3 — Reporting Summary [file 41467_2022_34877_MOESM3_ESM.pdf]

nature portfolio

Corresponding author(s): Jilong Yang

Last updated by author(s): Oct 23, 2022

Reporting Summary

Nature Portfolio wishes to improve the reproducibility of the work that we publish. This form provides structure for consistency and transparency in reporting. For further information on Nature Portfolio policies, see our Editorial Policies and the Editorial Policy Checklist.

Statistics

For all statistical analyses, confirm that the following items are present in the figure legend, table legend, main text, or Methods section.

n/a	Confirmed	
☐	☒	The exact sample size (*n*) for each experimental group/condition, given as a discrete number and unit of measurement
☐	☒	A statement on whether measurements were taken from distinct samples or whether the same sample was measured repeatedly
☐	☒	The statistical test(s) used AND whether they are one- or two-sided<br>*Only common tests should be described solely by name; describe more complex techniques in the Methods section.*
☐	☒	A description of all covariates tested
☐	☒	A description of any assumptions or corrections, such as tests of normality and adjustment for multiple comparisons
☐	☒	A full description of the statistical parameters including central tendency (e.g. means) or other basic estimates (e.g. regression coefficient) AND variation (e.g. standard deviation) or associated estimates of uncertainty (e.g. confidence intervals)
☐	☒	For null hypothesis testing, the test statistic (e.g. *F*, *t*, *r*) with confidence intervals, effect sizes, degrees of freedom and *P* value noted<br>*Give P values as exact values whenever suitable.*
☒	☐	For Bayesian analysis, information on the choice of priors and Markov chain Monte Carlo settings
☒	☐	For hierarchical and complex designs, identification of the appropriate level for tests and full reporting of outcomes
☐	☒	Estimates of effect sizes (e.g. Cohen's *d*, Pearson's *r*), indicating how they were calculated

Our web collection on statistics for biologists contains articles on many of the points above.

Software and code

Policy information about availability of computer code

Data collection	No specialized software was used for data acquisition
Data analysis	All software used in this study are published and cited either in the main text or Online method. Here is the list of software used in this study. CellRanger v2.1.1, R v3.6.2 , PyTorch v2.3.1, Scanpy v2.1, inferCNV v1.5.1, Miscell v1.0.0, ModiFit LT v3.2, and various R 3.6+ packages (e.g., pheatmap v1.0.12, reshape2 v1.4.4, monocle v2.14.0, survival v 3.1.12, ,Seurat v3.1.5, lmmunarch v 0.6.7, maftools v2.2.10, GSVA v1.34.0)

For manuscripts utilizing custom algorithms or software that are central to the research but not yet described in published literature, software must be made available to editors and reviewers. We strongly encourage code deposition in a community repository (e.g. GitHub). See the Nature Portfolio guidelines for submitting code & software for further information.

Data

Policy information about availability of data

All manuscripts must include a data availability statement. This statement should provide the following information, where applicable:

- Accession codes, unique identifiers, or web links for publicly available datasets
- A description of any restrictions on data availability
- For clinical datasets or third party data, please ensure that the statement adheres to our policy

The raw DNA sequencing, TCR sequencing and single-cell RNA sequencing data generated in this study have been deposited in the Sequence Read Archive (SRA) database under accession code PRJNA862451 (https://www.ncbi.nlm.nih.gov/sra/PRJNA862451). The raw bulk RNA sequencing data generated in this study have

been deposited in the Gene Expression Omnibus (GEO) database under accession code GSE215121 (https://www.ncbi.nlm.nih.gov/geo/query/acc.cgi?acc=GSE215121). The processed single-cell/bulk RNA data are available at the GEO database under accession code GSE215121. The remaining data generated in this study are provided in the Supplementary Information or Source Data file. The publicly data used in this study are available in the GEO database under accession code GSE72056 (https://www.ncbi.nlm.nih.gov/geo/query/acc.cgi?acc=GSE72056), GSE189889 (https://www.ncbi.nlm.nih.gov/geo/query/acc.cgi?acc=GSE189889), and GSE120575 (https://www.ncbi.nlm.nih.gov/geo/query/acc.cgi?acc= GSE120575).

Human research participants

Policy information about studies involving human research participants and Sex and Gender in Research.

Reporting on sex and gender	Patients with melanoma undergoing primary surgery for acral and cutaneous melanoma at Tianjin Medical University cancer hospital,China .We have provided individual level data of the gender and age and other clinical metadata in the Supplementary Data that accompany the manuscript.
Population characteristics	For scRNA-seq, all patients were acral and cutaneous melanoma, age ranging from 54-80y, patient characteristics are listed in Supplementary table 1.<br>For validation of the clinical cohort (TMCH-57 and TMCH-602): all patients characteristics are listed in Supplementary Data1.
Recruitment	Patients undergoing surgery for melanoma at Tianjin Medical University Cancer Hospital were approached for consent to donate research tissue for research. Patients had to have sufficient tumor volume to be able to support collection of extra tissue beyond what would be required for pathologic diagnosis. At surgical removal, a research pathologist evaluates if there is sufficient tissue that can be collected for research use without compromising diagnostic staging.<br>We recruited patients whose tumors were of sufficient size to enable sufficient collection of tumor aliquots that are to be dissociated for single cell analyses. This may have led to a selection bias towards tumors that are of sufficient size to enable analyses. This is inherent in single cell studies. The samples are mainly from Asian races and the sample size was limited. The characterization of acral melanoma in different ethnicities still needs to be further clarified by incorporating large sample sizes of different ethnicities.
Ethics oversight	All clinical specimens in this study were collected with informed consent for research use and were approved by the Tianjin Medical University Cancer Hospital institutional Review Boards in accordance with the Declaration of Helsinko, under protocol number bc2022110.

Note that full information on the approval of the study protocol must also be provided in the manuscript.

Field-specific reporting

Please select the one below that is the best fit for your research. If you are not sure, read the appropriate sections before making your selection.

☒ Life sciences ☐ Behavioural & social sciences ☐ Ecological, evolutionary & environmental sciences

For a reference copy of the document with all sections, see nature.com/documents/nr-reporting-summary-flat.pdf

Life sciences study design

All studies must disclose on these points even when the disclosure is negative.

Sample size	We present single-cell RNA-seq data from 7 acral and 4 cutaneous menlanoma from 6 acral and 3 cutaneous melanoma patients. We furthermore retrospectively analyzed 42 acral and 15 cutaneous melanoma samples of 57 patients and 602 clinical data from melanoma patients. Sample size was determined by the availability of patient samples.
Data exclusions	We excluded some patients with non malignant melanoma after verification by pathological experts
Replication	All reported findings were replicated across multiple independent biological samples.
Randomization	The patients were recruited randomly in this study. The sample that occurs in the palmar side of the extremities and is confirmed as melanoma by pathology is considered as acral melanoma, and the melanoma that occurs in other skin is considered as cutaneous melanoma.
Blinding	The researchers were blind to the clinical annotations at the point of biologic discovery.

Reporting for specific materials, systems and methods

We require information from authors about some types of materials, experimental systems and methods used in many studies. Here, indicate whether each material, system or method listed is relevant to your study. If you are not sure if a list item applies to your research, read the appropriate section before selecting a response.

Materials & experimental systems

n/a	Involved in the study
☐	☒ Antibodies
☒	☐ Eukaryotic cell lines
☒	☐ Palaeontology and archaeology
☒	☐ Animals and other organisms
☐	☐ Clinical data
☒	☐ Dual use research of concern

Methods

n/a	Involved in the study
☒	☐ ChIP-seq
☐	☒ Flow cytometry
☒	☐ MRI-based neuroimaging

Antibodies

Antibodies used

For immunofluorescence
anti-CD8A (1:200, CST, catalog number: 85336, Clone numbers: D8A8Y)
anti-CD4 (1:200, Abcam, catalog number:ab133616, Clone numbers: EPR6855)
anti-CD20 (1:200, Abcam, catalog number: ab78237, Clone numbers: EP459Y
anti-FOXP3 (1:200, CST, catalog number: 98377, Clone numbers: D2W8E™)
anti-TIM3 (1:200, Abcam, catalog number: ab241332, Clone numbers: EPR22241)
anti-PD1 (1:200, CST, catalog number: 86163, Clone numbers: D4W2J)
anti-GZMB (1:200, Abcam, catalog number: ab255598, Clone numbers: EPR22645-206)
anti-CCR7(1:200, Abcam, catalog number: ab32527, Clone numbers: Y59).
TSA (Absin Bioscience, catalog number: abs50013)
For Flow cytometry
anti-CD45 APC/Cyanine7(Biolegend, catalog number: 304014, Clone numbers: HI30)
FITC Annexin V Apoptosis Detection Kit I (BD PharmingenTM, catalog number: 556547)

Validation

For immunofluorescence
anti-CD8A (1:200, CST, catalog number: 85336, Clone numbers: D8A8Y)
-https://www.cellsignal.cn/products/primary-antibodies/cd8a-d8a8y-rabbit-mab/85336?site-search-type=Products&N=4294956287&Ntt=anti-cd8a&fromPage=plp
-Validated for immunohistochemistry (IHC) in paraffin-embedded human tissues by the manufacturer
anti-CD4 (1:200, Abcam, catalog number:ab133616, Clone numbers: EPR6855)
-https://www.abcam.cn/cd4-antibody-epr6855-ab133616.html
-Validated for immunohistochemistry (IHC) in paraffin-embedded human tissues by the manufacturer
anti-CD20 (1:200, Abcam, catalog number: ab78237, Clone numbers: EP459Y
-https://www.abcam.com/cd20-antibody-ep459y-ab78237.html
-Validated for iImmunocytochemistry/Immunofluorescence in paraffin-embedded human tissues by the manufacturer
anti-FOXP3 (1:200, CST, catalog number: 98377, Clone numbers: D2W8E™)
-https://www.cellsignal.cn/products/primary-antibodies/foxp3-d2w8e-rabbit-mab-ihc-specific/98377?site-search-type=Products&N=4294956287&Ntt=98377&fromPage=plp
-Validated for immunohistochemistry (IHC) in paraffin-embedded human tissues by the manufacturer
anti-TIM3 (1:200, Abcam, catalog number: ab241332, Clone numbers: EPR22241)
-https://www.abcam.com/tim-3-antibody-epr22241-ab241332.html
-Validated for immunohistochemistry (IHC) in paraffin-embedded human tissues by the manufacturer
anti-PD1 (1:200, CST, catalog number: 86163, Clone numbers: D4W2J)
-https://www.cellsignal.cn/products/primary-antibodies/pd-1-d4w2j-xp-rabbit-mab/86163?site-search-type=Products&N=4294956287&Ntt=86163&fromPage=plp&_requestid=4573310
-Validated for immunohistochemistry (IHC) in paraffin-embedded human tissues by the manufacturer
anti-GZMB (1:200, Abcam, catalog number: ab255598, Clone numbers: EPR22645-206)
-https://www.abcam.com/granzyme-b-antibody-epr22645-206-ab255598.html
-Validated for immunohistochemistry (IHC) in paraffin-embedded human tissues by the manufacturer
anti-CCR7(1:200, Abcam, catalog number: ab32527, Clone numbers: Y59)
-https://www.abcam.com/ccr7-antibody-y59-ab32527.html
-Validated for Immunocytochemistry/Immunofluorescence in paraffin-embedded human tissues by the manufacturer
TSA (Absin Bioscience, catalog number: abs50013)
-https://www.absin.cn/five-color-multi-label-immunofluorescence-kit/abs50013.html
For Flow cytometry
anti-CD45 APC/Cyanine7(Biolegend, catalog number: 304014, Clone numbers: HI30)
-https://www.biolegend.com/en-us/products/apc-cyanine7-anti-human-cd45-antibody-1914
-Validated for Flow Cytometric in human peripheral blood lymphocytes by the manufacturer
FITC Annexin V Apoptosis Detection Kit I (BD PharmingenTM, catalog number: 556547)
-https://www.bdbiosciences.com/zh-cn/products/reagents/flow-cytometry-reagents/research-reagents/panels-multicolor-cocktails-ruo/fitc-annexin-v-apoptosis-detection-kit-i.556547
-Validated for Flow Cytometric in human T-cell leukemia by the manufacturer

Clinical data

Policy information about clinical studies
All manuscripts should comply with the ICMJE guidelines for publication of clinical research and a completed CONSORT checklist must be included with all submissions.

Clinical trial registration: 452 TCGA SKCM clinical data and 602 melanoma patients'clinical data from TMCH

Study protocol: We applied the survival data of TCGA patients to study the differences between the signatures and prognosis of 5 functions of tumor cells . The clinical data of melanoma patients from TMCH were used to compare the survival differences of two melanoma subtypes

Data collection: The clinical data ofTCGA comes from UCS C Xena database, and the clinical data of TMCH comes from the database ofTianjin Medical University Cancer Hospital.

Outcomes: We want to further elaborate the genomic mechanism behind clinical differences by connecting the results found in single cell data with the clinical information of patients.

Flow Cytometry

Plots

Confirm that:

☒ The axis labels state the marker and fluorochrome used (e.g. CD4-FITC).

☒ The axis scales are clearly visible. Include numbers along axes only for bottom left plot of group (a 'group' is an analysis of identical markers).

☒ All plots are contour plots with outliers or pseudocolor plots.

☒ A numerical value for number of cells or percentage (with statistics) is provided.

Methodology

Sample preparation: 1 acral melanoma samples and 1 cutaneous melanoma samples

Instrument: BD FACS Aria II

Software: BD FACS Diva Software, ModFit LT 3.2

Cell population abundance: We sorted out CD45 negative tumor cells in the co-culture of tumor cells and CD8 +T cells by CD45,. Then PI-Annexin V was used to detect the apoptosis ratio of tumor cells.

Gating strategy: Description of Gating strategy was presented in legend of Supplementary figure 10.

☒ Tick this box to confirm that a figure exemplifying the gating strategy is provided in the Supplementary Information.
